# L’offre des services de vaccination en milieu urbain, au Cameroun: étude de cas du District de Santé de Djoungolo

**DOI:** 10.11604/pamj.2016.25.213.8803

**Published:** 2016-12-06

**Authors:** Armelle Viviane Ngomba, Basile Kollo, André Fouda Bita, Fabrice Nembot Djouma, Jean Marie Edengue, Marie Josée Elongue, Dieudonné Adiogo

**Affiliations:** 1 Faculté de Médecine et des Sciences Pharmaceutiques, Université de Douala Cameroun; 2 Département de Sciences Biomédicales, Université de Dschang, Cameruon, PO Box 067, Dschang, Cameroun; 3 Agence de Médecine Préventive, Yaoundé, Cameroun; 4 Ministère de la Santé Publique, Cameroun

**Keywords:** Offre des services, vaccination, urbain, Cameroun, Service programs, immunization, urban, Cameroon

## Abstract

**Introduction:**

Les métropoles camerounaises concentrent un nombre croissant d'enfants cibles, non atteints par les services de vaccination de routine.

**Méthodes:**

Une étude transversale descriptive, basée sur échantillonnage exhaustif des formations sanitaires légales offrant en routine des services de vaccination a été réalisée dans le district de santé de Djoungolo, (ville de Yaoundé). L'évaluation de l'offre des services de vaccination a été calquée sur l'approche « Atteindre Chaque District ».

**Résultats:**

Des 70 formations sanitaires ayant participé à l'étude, 3 (4,3%) disposaient d'un micro plan actualisé pour la vaccination de routine. Des 63 (89,4%) formations sanitaires possédant un réfrigérateur fonctionnel, 12 (19,0%) vaccinaient au quotidien en stratégie fixe. Cinquante-sept (81,0%) formations sanitaires ne conduisaient pas des sessions de vaccination en stratégies avancées. La participation des membres de la communauté aux activités de vaccination de routine étaient effective dans 1 aire de santé sur les 12 que compte le district. Une courbe de suivi des couvertures vaccinales correctement renseignée étaient disponible dans 6 (8,5%) formations sanitaires.

**Conclusion:**

L'approche « Atteindre Chaque District » telle que mise en œuvre dans le district de santé de Djoungolo limite l'atteinte d'un maximum d'enfants cibles. L'effectivité d'une micro planification réaliste, la régularité des sessions de vaccination en stratégie fixes et avancées, le suivi des données orienté vers l'action, la redynamisation communautaire en faveur de la vaccination sont des voies d'amélioration des prestations des services de vaccination, dans ce district.

## Abstract (en)

**Introduction:**

Cameroon's cities have a growing concentration of target children not adequately covered by routine immunization programmes.

**Methods:**

We conducted a descriptive cross-sectional study, based on exhaustive sampling of legal health care facilities offering routine immunization services in the health district of Djoungolo (city of Yaoundé). The evaluation of the immunization programmes was based on the “Reaching Every District” approach.

**Results:**

Out of the 70 health care facilities that participated in the study, 3 (4.3%) had an updated microplan for routine immunization. Out of the 63 (89.4%) health care facilities with functional refrigerator, 12 (19.0%) vaccinated employing a fixed strategy on daily basis. Fifty-seven (81.0%) health care facilities did not conduct immunization sessions employing advanced strategy. Community participation in routine immunization programs was effective in 1 out of 12 districts health areas. A steadily updated vaccine tracking curve was available in 6 (8.5%) health facilities.

**Conclusion:**

The “Reaching Every District” approach implemented in Djoungolo health care district doesn't provide adequate coverage of a maximum rate of targeted children. The effectiveness of realistic microplanning, the regularity of vaccination sessions employing fixed and advanced strategies, action-oriented data monitoring, community revitalization plan to promote immunization are ways to improve the provision of vaccination services in this district.

## Introduction

La vaccination est l'une des interventions de santé publique les plus efficaces et efficientes pour la réduction de la mortalité et morbidité infanto-juvénile dans le monde. Grâce à elle, l'incidence de la poliomyélite a été réduite de 99%; de même l'incidence et la mortalité attribuable à la diphtérie, la coqueluche, la rougeole, le tétanos, et les méningites à Méningocoque ont considérablement été abaissées [1, 2]. Le Cameroun est un pays d'Afrique Centrale dont la population totale est estimée 17 463 836 habitants [3]. Les couvertures vaccinales des enfants de 0-11 mois y ont été croissantes au cours de la dernière décennie, passant de 73% en 2003 à 89% en 2013, pour l'antigène traceur (DTP3) [4]. En 2014, il a été observé une tendance à la baisse de ces couvertures; également, une recrudescence des épidémies de poliomyélite, et de rougeole au sein de la population infanto juvénile [4, 5]. De l'analyse de la situation faite par le Programme Elargi de Vaccination (PEV), il ressort que 30% des enfants cibles manqués par les services de vaccination de routine résidaient à Yaoundé ou à Douala, les deux métropoles du pays. [6]. En s'appuyant sur le modèle de l'approche « Atteindre Chaque District » [7, 8], l'objectif de la présente étude était d'évaluer l'offre des services de vaccination en milieu urbain.

## Méthodes

**Type d'étude:** il s'agissait d'une étude transversale descriptive, basée sur des interviews du personnel de santé, l'observation des équipements de la chaîne du froid et une revue des documents de gestion du PEV. L'échantillonnage a été exhaustif, incluant ainsi toutes les formations sanitaires (FS) légales (publiques et privées), offrant des services de vaccination en routine dans le district étudié.

**Lieu d'étude:** l'étude s'est déroulée de Février à Mai 2014 dans le district de santé de DJOUNGOLO. Ce dernier est situé au cœur de la ville de Yaoundé, et y abrite 30% de la population. Cette population est essentiellement jeune, caractérisée par une diversité ethnique, religieuse et des migrations importantes liées aux activités professionnelles formelles ou informelles [3]. Il est subdivisé en 12 aires de santé dans lesquelles sont reparties 72 formations sanitaires légales offrant des prestations de vaccination en routine. Pour une cible annuelle estimée en 2013 à 27 775 enfants de 0-11 mois, la couverture vaccinale (CV) en DTC3 (antigène traceur) au sein du district était de 68% [9]. Entre les aires de santé de ce district, la couverture vaccinale variait entre 32% et 131% (Tableau 1).

**Tableau 1:** Distribution de la population, des services de vaccination et des couvertures vaccinales dans le district de santé de Djoungolo

Aire de santé	Pop totale estimée en 2013	Enfants 0-11 mois	Nombre de FS qui vaccine	CV en DTC3 en 2013
Elig-essono	28 523	1 141	2	32%
Emana	55 728	2 229	8	105%
Essos	70 541	2 822	4	56%
Etoa meki	48 614	1 945	2	63%
MballaII	73 823	2 953	4	67%
MballaV	87 123	3 485	13	83%
Mvog ada	82 993	3 320	5	131%
Nfandena	92 004	3 680	5	55%
Nkolmesseng	39 130	1 565	8	98%
Nkolomdom	40 874	1 635	6	118%
Stinga village	39 875	1 595	9	42%
Nlongkak	35 124	1 405	6	92%
**Total DJOUNGOLO**	**694 372**	**27 775**	**72**	**68%**

Source: Délégation Régionale du Centre. Rapport d’activité Décembre 2013

**Collecte et analyse des données:** les données collectées ont été consignées sur une fiche de synthèse, puis analysées à l'aide du logiciel SPSS. Le plan d'analyse (Tableau 2) a été inspiré des indicateurs de suivi recommandés par l'OMS dans l'approche « Atteindre Chaque District » [7]. Cette approche repose sur cinq composantes qui sont: i) la planification pour la vaccination de routine et la gestion des ressources de vaccination; **ii)** les liens entre les services de vaccination et la communauté; **iii)** les prestations des services de vaccination; **iv)** la mise en œuvre des supervisions formatives; **v)** le « monitoring pour action » [7].

**Tableau 2:** Plan d’analyse des informations collectées

Composantes de l’approche ACD	Indicateurs de suivi
Planification pour la vaccination de routine et gestion des ressources	% des formations sanitaires (FS) qui disposaient d’un micro plan de vaccination de routine actualisé pour la période couvrant l’enquête.
	% des FS qui disposaient d’un réfrigérateur fonctionnel et d’une source d’énergie électrique
	% des FS qui n’ont pas connu une rupture de stock en vaccins (tout antigènes confondus) au cours de la période de l’enquête, ou les trois mois précédents.
	% des FS disposant d’au moins un agent de santé formé pour la vaccination de routine.
Liens entre les services de vaccination et les communautés	% des aires de santé dans lesquelles les organisations à base communautaires sont impliquées dans la vaccination de routine;
Prestations des services de vaccination	% des FS qui vaccinent au quotidien au cours de la période d’enquête
	% de FS qui effectuent des stratégies avancées au moins une fois par mois. cours de la période d’enquête.
Supervision formative	% des FS qui ont bénéficié d’au moins une supervision formative axé sur la vaccination de routine au cours de la période de l’enquête ou les trois mois précédents.
Mécanisme de suivi- évaluation (monitoring pour action)	% des FS qui disposent de diagrammes actualisés de suivi de la vaccination, correctement dessinés ET affichés. au cours de la période d’enquête.

**Considérations éthiques:** l'étude a été réalisée avec l'accord du comité d'éthique institutionnel de l'Université de Douala, l'autorisation des autorités administratives compétentes et le consentement éclairé des personnes interviewées. Pour des raisons de confidentialité, les noms des formations sanitaires enquêtées ne sont pas mentionnés dans cet article.

## Résultats

Sur un total de 72 formations sanitaires éligibles, 70 ont accepté de participer à l'enquête; soit un taux de réponses de 97%.

### Planification, gestion des ressources et participation communautaire

Un micro plan actualisé pour la vaccination de routine a été retrouvé dans 3 (4,3%) formations sanitaires enquêtées. Il est ressortit des entretiens avec le personnel en charge de la vaccination que le manque d'intérêt pour l'élaboration de des micro plans est principalement due à l'insuffisance des ressources humaines et financières nécessaires pour leur mise en œuvre. Dans 69 (98,6%) formations sanitaires enquêtées, la principale source d'énergie était l'électricité tandis qu'une (1,4%) une formation sanitaire utilisait de l'énergie solaire. Des 63 (89,4%) formations sanitaires dotées d'un réfrigérateur fonctionnel, 40 (63,5%) disposaient d'une fiche de suivi des températures de conservation des vaccins correctement renseignée au cours des trois mois précédent l'enquête. Les ruptures de stocks en vaccins, tout antigène confondu ont été relevées dans 39 (55,7%) formations sanitaires au cours des trois mois précédents l'enquête (Tableau 3). Toutes les formations sanitaires enquêtées disposaient d'au moins d'un personnel en charge de la vaccination; ce personnel était polyvalent et dans 73% des cas, avait suivi au moins un renforcement des capacités relatif aux prestations de la vaccination de routine. La communauté à travers les structures de dialogue avait participé à la planification, la mise en œuvre, le suivi ou l'évaluation des activités de vaccination de routine dans une aire de santé (8,3%) sur les douze que compte le district.

**Tableau 3:** Distribution des formations sanitaires en fonction de la continuité du stock en vaccins

Aires de santé (AS)	Nombre de FS dans l’aire	Au moins une rupture de stocks de vaccins (tout antigène confondue) pendant l’enquête ou au cours de 03 mois précédents			
		Oui		Aucune	
		Nombre de FS	Fréquence (%)	Nombre de FS	Fréquence (%)
Elig Essono	2	1	50,0	1	50,0
Emana	8	2	25,0	6	75,0
Etoa Meki	1	0	0,0	1	100,0
Mballe Ii	4	2	50,0	2	50,0
Mballa V	13	6	46,2	7	53,8
Tsinga Village	9	6	66,7	3	33,3
Essos	4	2	50,0	2	50,0
Mfandena	5	2	40,0	3	60,0
Nkolmeseng	7	5	71,4	2	28,6
Mvog Ada	5	4	80,0	1	20,0
Nlongkak	6	4	66,7	2	33,3
Nkolondom	6	5	83,3	1	16,7
**Total**	70	39	55,7	31	44,3

P= 0,538

### Prestations des services de vaccination

Les sessions de vaccination en stratégie fixe étaient organisées à un rythme hebdomadaire dans 49 (70,0%) formations sanitaires enquêtées. Des 63 (89,4%) formations sanitaires dotées un réfrigérateur fonctionnel, 12 (19,0%) vaccinaient au quotidien en stratégie fixe. Le taux de réalisation des sessions de vaccination en stratégies avancées planifiées au cours des trois derniers mois précédant l'enquête était de 4,5%. Des 70 formations sanitaires enquêtées, 57 (81,4%) ne menaient aucune stratégie avancée. Le personnel en charge de la vaccination interviewé a justifié l'irrégularité dans l'organisation des séances de vaccination en stratégie fixe comme une mesure visant à minimiser les taux de perte des vaccins. Par ailleurs, le faible taux de réalisation des stratégies avancées a été justifié par l'insuffisance du matériel roulant et des mesures incitatives;

### Supervision formative et suivi pour l'action

Des 70 formations enquêtées 41 (58,6%) ont été supervisées au moins une fois au cours des trois derniers mois précédant l'enquête (Tableau 4). De ces 41 formations sanitaires, 8 (19,5%) disposaient d'un plan de résolution des problèmes issus des dites supervisions. Une courbe de suivi des couvertures vaccinales correctement renseignée était disponible dans 6 (8,6%) des formations sanitaires enquêtées. Des entretiens avec le personnel de santé il ressort que l'enjeu du rapportage correct et de l'analyse régulière des données de vaccination n'était pas suffisamment compris par les responsables des formations sanitaires.

**Tableau 4:** Distribution des formations sanitaires ayant bénéficié d’une supervision formative au cours des 3 derniers mois précédant l’enquête

Aires de santé (AS)	Nombre de FS dans l’aire	Proportion des formations sanitaires ayant bénéficié d'au moins une supervision formative (%)
Elig Essono	2	50,0
Emana	8	62,5
Etoa meki	1	100,0
Mballe II	4	75,0
Mballa V	13	69,2
Tsinga Village	9	33,3
Essos	4	50,0
Mfandena	5	80,0
Nkolmeseng	7	85,7
Mvog Ada	5	20,0
Nlongkak	6	50,0
Nkolondom	6	50,0
Total	70	58,6

## Discussion

De cette étude, dont le but était d'évaluer l'offre des services de vaccination dans le district de santé de Djoungolo, il ressort que malgré une source d'électricité régulière, une couverture en réfrigérateurs fonctionnels de 89%, et un personnel disponible, les séances de vaccination étaient organisées à un rythme hebdomadaire et exclusivement en stratégie fixe dans plus de 70% des formations sanitaires. En outre, la participation des membres de la communauté et le suivi des couvertures vaccinales étaient faibles.

### Planification, gestion des ressources et participation communautaire

La micro planification est fondamentale pour identifier les besoins et prioriser les actions spécifiques à mettre en œuvre pour améliorer les couvertures vaccinales [7, 8]. Une étude réalisée au Cameroun en 2004 a établie l'existence d'une association entre la disponibilité de micro plan de vaccination au niveau des formations sanitaires et la couverture vaccinale [10] En ce sens, le manque d'intérêt pour la micro planification et la faible participation de la communauté dans cet exercice constituaient à DJOUNGOLO, le premier goulot d'étranglement du système local de vaccination. Le PEV devrait veiller annuellement à l'actualisation des micros plans et accompagner les responsables du district et des aires de santé dans cet exercice. La proportion de 98,6% des formations sanitaires régulièrement approvisionnées en électricité tient de la localisation urbaine du district et corrobore les statistiques nationales en matière d'électrification dans le pays [11]. Par contre, l'estimation de la disponibilité des réfrigérateurs fonctionnels dans les formations était de 17 à 25% supérieure à la proportion révélée par d'autres études réalisées dans le pays [12–14]. Cet écart pourrait s'expliquer par la non différenciation lors du dénombrement, des réfrigérateurs homologués (PQS) par l'OMS et les réfrigérateurs domestiques peu recommandés pour la conservation des vaccins mais fréquemment retrouvés dans les formations sanitaires enquêtées, ceux-ci étant vendu sur le marché local et à des prix accessibles. Afin de conserver leur efficacité, les vaccins doivent être conservés entre +2 et +8 degrés Celsius, au niveau des formations sanitaires. Cette température de conservation doit être continuellement suivie pour prévenir la détérioration des vaccins par la chaleur ou la congélation. Cette étude relève des irrégularités dans le suivi des températures de conservation des vaccins; fait plus tôt courant dans le pays, explicitant ainsi un des inconvénients de la méthode manuelle de relevé des températures [13–15]. Les ruptures de stocks en vaccins observées dans les formations sanitaires lors de l'enquête pourraient majorer les occasions manquées de vaccination. Toutefois, selon une étude randomisée réalisée dans 80 formations sanitaires réparties dans 51 districts de santé au Cameroun, il n'a pas été établi une association statistiquement significative entre la continuité des stocks des vaccins au niveau opérationnel et les couvertures vaccinales [10].

En plus du leadership local et des compétences managerielles des responsables du district, la disponibilité des prestataires des soins, leur répartition équitable et leur motivation constituent des facteurs de bonnes performances des services de vaccination [16, 17]. Au Cameroun, le personnel de santé disponible au niveau opérationnel est en général insuffisant et démotivé [18, 19]. En outre, la vaccination de routine n'étant pas une activité génératrice de revenu, les responsables des formations sanitaires manquent d'engagement pour adapter les prestations des services de vaccination aux spécificités de leurs communautés [20]. Cette dernière raison explique davantage les faibles couvertures vaccinales enregistrées par le district de santé de DJOUNGOLO, malgré la disponibilité dans les formations sanitaires d'un personnel formé pour les prestations de vaccination de routine. La qualité de l'interface entre la communauté et les services de vaccination est un des déterminants directs de la couverture vaccinale [7, 16, 21]. Les membres de la communauté peuvent être mis à contribution pour faciliter l'adhésion de la population à la vaccination, retrouver les perdus de vue, apporter un appui logistique au service de vaccination. Dans le district de santé de DJOUNGOLO le potentiel communautaire n'était pas suffisamment exploité pour améliorer les couvertures vaccinales. Ce déficit relevait principalement d'une faible dynamique et capacitation des structures de dialogues susceptibles de jouer un rôle catalyseur pour le Programme Elargi de Vaccination. En outre, il se pose la question de la motivation des travailleurs communautaires [22].

### Prestations des services de vaccination

L'irrégularité des séances de vaccination en stratégies fixes et avancées observées dans le district de santé de Djoungolo concernent au moins 50% des formations du pays. Il s'agit d'un problème récurrent résultant en général d'une faible micro planification, et d'une faible disponibilité des ressources (financières, humaines, logistique) [20]. Le district de santé de DJOUNGOLO à l'instar de plusieurs localités urbaines des pays en voie de développement hébergent une proportion importante d'enfants cibles difficiles d'accès du fait des barrières socioculturelles [23, 24]. L'inadéquation des sessions de vaccination en stratégie fixe, la quasi absence des sessions de vaccination en stratégies avancées et la faible participation des membres de la communauté agissent en synergie pour limiter l'utilisation des services de vaccination par les populations vulnérables. [23, 24].

### Supervision formative et suivi des données pour l'action

Bien que n'étant pas l'unique déterminant, la régularité dans le suivi des données et la supervision formative du personnel est positivement associée à de meilleures couvertures vaccinales [10, 16]. Dans le district de santé de DJOUNGOLO, la supervision du personnel et le suivi des données de vaccination étaient irrégulières, limitant ainsi l'assurance qualité des prestations et des données de vaccination. L'insuffisance des supervisions est observée dans l'ensemble du territoire et touche aussi bien le niveau opérationnel, intermédiaire et central et entretien une faible redevabilité du personnel de santé [20].

### Limites de l'étude

Les résultats de cette étude présentent la situation de l'organisation des services vaccination dans un district de santé urbain au Cameroun. Ne se limitant qu'à l'étude d'un cas, les tendances relevées devraient être confirmées par une évaluation à plus large échelle dans les métropoles camerounaises. D'autres facteurs de performances du Programme Elargi de Vaccination tels que l'attitude des populations vis-à-vis des services de vaccination offerts, la capitalisation des appui techniques et financiers des organisations non gouvernementales, les capacités managerielles et le leadership des responsables des district de santé n'ont pas été abordé dans le cadre de cette étude, mais leur analyse permettra d'identifier les points d'actions pouvant contribuer à améliorer durablement les couvertures vaccinales au Cameroun.

## Conclusion

L'approche « Atteindre Chaque District » telle que mise en œuvre dans le district sanitaire de Djoungolo limite l'atteinte d'un maximum d'enfants cibles. L'effectivité d'une micro planification réaliste, la conduite régulière des sessions de vaccination en stratégies fixes et avancées, le suivi et la supervision orientés vers l'action, et la participation communautaire en faveur de la vaccination sont des voies d'amélioration. La couverture vaccinale étant un des indicateurs clés de performance du système local de santé, la situation décrite par cette étude suscitent des interrogations sur l'adéquation des ressources dont disposent les structures de santé au niveau opérationnel pour produire les résultats escomptés, ainsi que sur l'application des principes de redevabilité.

### Etat des connaissances actuelle sur le sujet

- L'approche Atteindre Chaque District (ACD) a été mis au point par l'OMS et ses partenaires en 2002, pour augmenter et maintenir des couvertures vaccinales de routines à un haut niveau au sein d'une communauté;
- Depuis lors, la plupart des pays africains y compris le Cameroun ont implémenté cette approche avec succès;
- Toutefois les résultats et la pérennité de la mise en œuvre de cette approche sont conditionnés par des facteurs contextuels spécifiques à chaque pays, tels que les ressources humaines, le leadership, l'engagement des autorités locales, la coordination.

### Contribution de notre étude à la connaissance

- Cette étude donne des informations actualisées sur les facteurs déterminants la faible atteinte des enfants ciblés par les services vaccination dans la principale métropole camerounaise, Yaoundé;
- Le non-respect des principes de l'approche ACD notamment, la micro planification réaliste, la conduite régulière des sessions de vaccination en stratégies fixes et avancées, le suivi et la supervision orientés vers l'action, la participation communautaire;
- La faible motivation du personnel de santé et la faible application des principes de redevabilité.
